# Adult nondysraphic cervicomedullary intramedullary lipoma: A case report and literature review

**DOI:** 10.1002/ccr3.7239

**Published:** 2023-04-24

**Authors:** Faramarz Roohollahi, Vahid Sharifi, Setare R. abousaeidi, Morteza F. Jouibari

**Affiliations:** ^1^ Department of Neurosurgery Shariati Hospital Complex, Tehran University of Medical Sciences Tehran Iran; ^2^ Spine center of excellence Yas hospital, Tehran University of Medical Sciences Tehran Iran

**Keywords:** intramedullary mass, nondysraphic, spinal lipoma

## Abstract

Intra‐dural perimedullary lipomas involving craniovertebral region are rare. There is no clear tumor‐cord border and patients are at high risk of neurological deficits after surgery. Partial resection of the mass and neural decompression are the main surgical strategies in symptomatic patients.

## Abstract 2

Spinal intramedullary lipoma within the craniovertebral region is extremely rare. These lesions are often associated with spinal dysraphism. Here, we present a nondysraphic one. Our patient was a 56‐year‐old woman who presented with progressive right shoulder pain and gait disturbance. Imaging showed an intradural lipoma, adherent to medulla, and upper cervical spinal cord. The patient underwent limited suboccipital craniotomy and C1‐C5 laminectomy. Lipoma was resected partially due to the adherence of the tumor to neural tissue. Her weakness was exacerbated immediately after surgery but improved within 4 weeks.

## 1 INTRODUCTION

Intramedullary spinal lipomas and especially in craniocervical area are extremely rare. They account for <1% of spinal cord tumors.^1^ The cervicothoracic and thoracic regions of the dorsal cord are most commonly involved.^2^ Clinical presentation is not distinctive, but radiographic evaluations can be very diagnostic.^3^ Usually, total resection is not possible, and safe subtotal resection is recommended.^2^

## 2 CASE PRESENTATION

The patient was a 56‐year‐old woman. She had suffered chronic but progressive pain in her right shoulder since 20 years ago. She also complained of clumsiness in her right upper and lower limbs, and gait disturbance started 2 months ago. She had no sensory loss or bowel/bladder involvement. In the physical examination, four limbs tone and strength were normal except mild weakness in right shoulder abduction. Light touch, pinprick, and vibration perception were normal on both sides. Deep tendon reflexes were hyperactive on the right side (3+), and Hoffman sign was present on the right side. Plantar responses were upward bilaterally. Routine laboratory data were normal.

**FIGURE 1:**
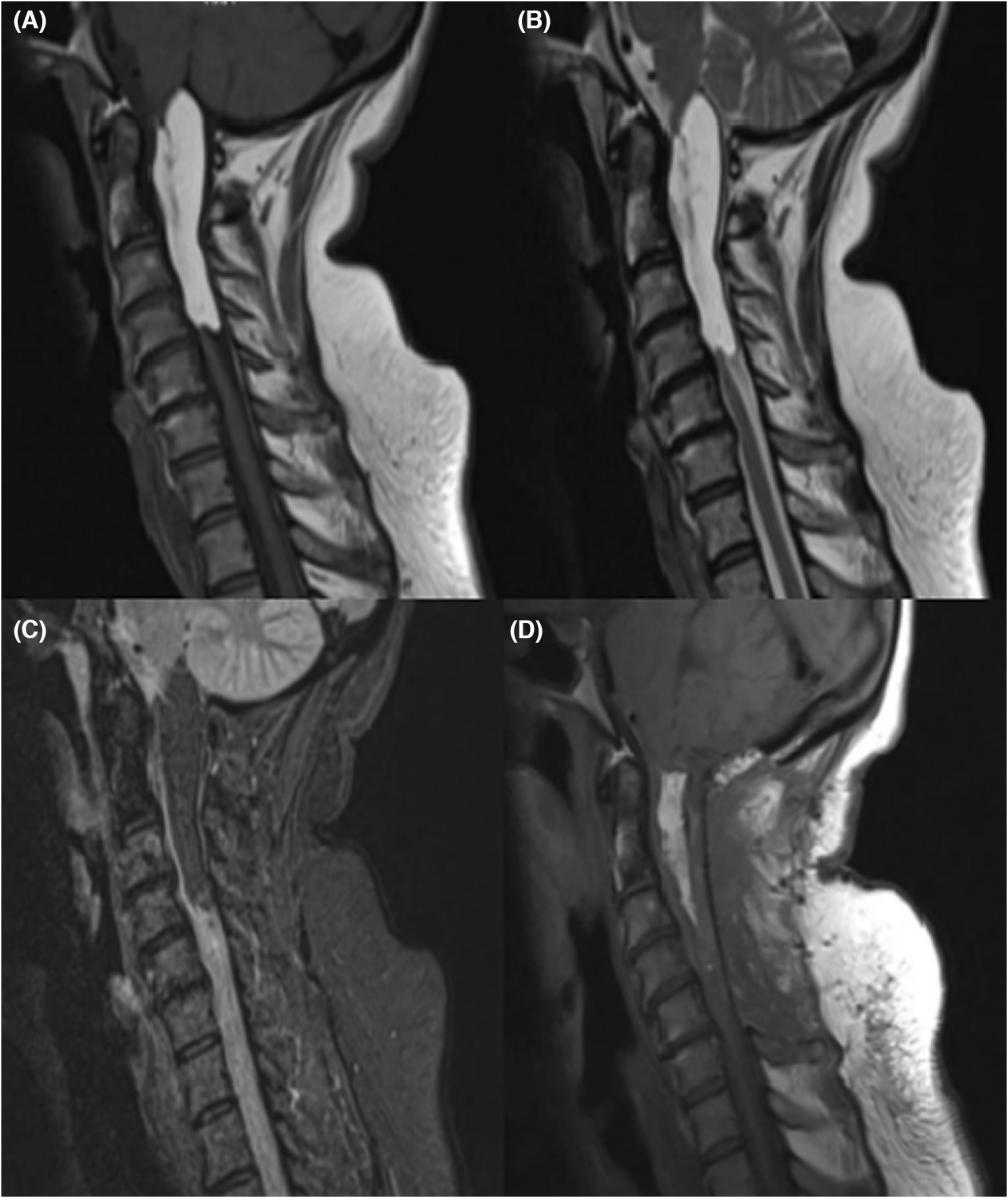
(A) Sagittal T1‐weighted view of the craniocervical region showing hyperintense perimedullary lesion. (B) Sagittal T2‐weighted view of the craniocervical region showing hyperintense perimedullary lesion. (C) Sagittal fat‐suppression sequence of the craniocervical region showing an intensity reduction in the lesion. (D) Post‐operation T1‐weighted MRI is showing subtotal resection of the lesion.

MRI showed an intradural well‐defined mass lesion extending from the medulla to C4 level. it was hyperintense in T1 and T2 sequences. Fat‐suppression sequence showed intensity reduction in the lesion in favor of fat tissue (Figure 1). No bone defect or deformity was seen in the cervical spine.

**FIGURE 2:**
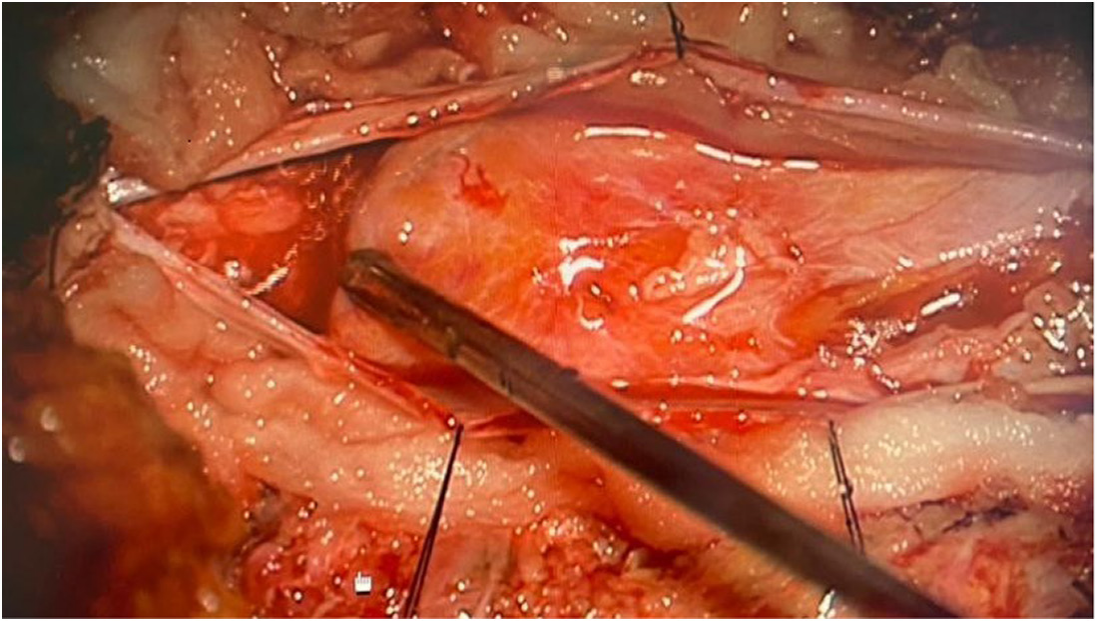
Intraoperative view of yellowish mass after durotomy.

Surgical excision was planned. A minimal suboccipital craniectomy and a wide C1 to C5 laminectomy were done in the prone position. The dura appeared normal. The dura and arachnoid opened in separate layers. Using microscope view, a large subpial yellowish mass from the dorsal medulla to the C4 level was evident (Figure 2). The tumor was resected gradually using the Cavitron ultrasonic aspirator (CUSA). Bleeding was controlled using bipolar cautery. Even after mass reduction, the tumor‐cord border was not identifiable except in superior and inferior poles. Due to tumor adherent features and neuromonitoring warnings, we stopped after approximately 70% tumor resection. Pathological findings were consistent with lipoma diagnosis.

Immediately after surgery, all four extremities were weakened, but the weakness was more severe on the right side. The shoulder pain decreased. Her weakness improved within 4 weeks of rehabilitation, and she could walk without help.

## 3 DISCUSSION

Spinal lipomas account for <1% of spinal cord lesions.^4^ They often present extramedullary in the lumbosacral region and are associated with spinal dysraphism.^5^, ^6^ These tumors are considered congenital lesions and are more common in childhood.^7^ Although adipocyte migration dysregulation in the embryonic period is a popular mechanism for describing dysraphic spinal cord lipomas, there is no acceptable theory explaining nondysraphic lipoma pathogenesis.^8^ Here, we report a nondysraphic lipoma with the intradural perimedullary presentation, which is extremely rare.

As symptoms are not specific and the disease course is indolent, diagnosis can be delayed. Symptoms improve after proper treatment except for severe deficits, which necessitate early detection and treatment.^9^ It seems that severe deficits are not the result of mass effect but they are caused by replacing normal neural tissue with fat tissue.^1^

We reviewed 13 case reports concerning intradural spinal lipoma within the craniovertebral junction, including our patient (Table 1). The male/female ratio is 10:3. Most patients are young adults. The functional improvement had been reported in eight cases. One regrowth has been reported, but reoperation was not needed.^10^ It seems that the clinical course of these tumors after surgery is indolent, and regrowth will not be a concern, although close follow‐up with MRI is recommended.

**TABLE 1:** Summary of adult patients with cervicomedullary intramedullary lipoma in the literature and present study

Authors and years	Age/sex	Level of the lesion	Preoperative symptoms	Duration of symptoms	Resection percent	Postoperative condition	Tumor regrowth	Follow‐up period
Drapkin et al^13^ (1974)	24 years/F	FM to C3	Quadriparesis	24 years	Subtotal	No functional improvement	No	3 months
Fan et al^14^ (1989)	18 years/M	FM to C5/C6	Quadriparesis	2 year	Subtotal	No functional improvement	No	4 months
Kodama et al^15^ (1991)	30 years/M	FM to C2	Quadriparesis	30 years	Subtotal (50%)	Functional improvement	No	1 month
Yamashita et al^16^ (1992)	37 years/M	FM to C3	Brown‐Sequard syndrome	5 years	Subtotal (40%)	Functional improvement	No	6 months
Feuvre et al^17^ (2004)	18 years/F	FM to C3	Quadriparesis/incontinence	14 months	Subtotal	Functional improvement	No	1 year
Yun et al^18^ (2007)	30 years/M	Medulla to C1	Dizziness/gait disturbance/headache	2 days	Subtotal	Decline functional status	NA	NA
Chagla et al^19^ (2008)	18 years/M	FM to C6	Quadriparesis	1 month	Subtotal	Functional improvement	No	18 months
Mohindra et al^20^ (2009)	20 years/M	FM to C2	Quadriparesis	6 months	Subtotal	Functional improvement	NA	NA
Khurana et al^21^ (2010)	20 years/M	Medulla to C5	Shoulder pain/upper extremities weakness	4 months	Subtotal	Functional improvement	No	2 months
Meher et al^12^ (2017)	30 years/M	Medulla to C4	Left shoulder and forearm weakness	2 months	Subtotal	No functional improvement	NA	NA
Iplikcioglu et al^10^ (2019)	28 years/M	Medulla to C2	Upper extremities weakness	3 months	Subtotal	Functional improvement	Regrowth at 2 years then stable in 8 years	8 years
Inoue et al^7^ (2019)	60 years/M	Medulla to C2	Right extremities weakness	3 months	Subtotal	Functional improvement	No	12 months
Faghih et al (2022) (present study)	56 years/F	Medulla to C4	Right shoulder pain/Gait disturbance	20 years	Subtotal (70%)	No functional improvement	No	1 month

Abbreviations: F, female; FM, foramen magnum; M, male; NA, not applicable.

Age is a major predicting factor of disease progression. The risk of progression for adult patients with dysraphic and nondysraphic spinal lipomas is low.^11^ MRI is diagnostic in most cases as fat is hyperintense in both T1 and T2 sequences and will be suppressed using fat‐suppression sequences.^12^

Management of these lesions is still controversial, and a patient‐specific approach is needed. The treatment goal is to stop neurological deterioration. Because of the significant risk of neurologic deterioration following surgery (like our patient), preventive surgery is not recommended.^1^

Although the amount of resection has increased using CUSA and microsurgical techniques under microscope,^22^ gross total resection is not advised because fat tissue is intermingled with neural tissue, and the tumor‐cord border is not clear.^9^

## 4 CONCLUSION

Nondysraphic perimedullary spinal lipomas are extremely rare. There is no consensus regarding the management of these lesions. Generally, trying for gross total resection can lead to severe neurological deficits and is not recommended. Subtotal tumor resection using CUSA under intraoperative neuromonitoring is a rational approach for most symptomatic cases.

## AUTHOR CONTRIBUTIONS

**Faramarz Roohollahi:** Conceptualization; methodology; writing – original draft. **Vahid Sharifi:** Data curation; methodology; validation; visualization. **Setare Rostami:** Investigation; visualization; writing – review and editing. **Morteza Faghih Jouibari:** Methodology; project administration; supervision; writing – review and editing.

## FUNDING INFORMATION

All the authors involved in this article are researchers with educational targets in medical universities. The authors are not funding by any corporation or government. Also, we have to clarify that all the writers involved in this article are researchers with educational targets in medical universities, and they have no government involvement or any other official representative.

## CONFLICT OF INTEREST STATEMENT

The authors report that there is no competing interest to declare.

## CONSENT

Written informed consent was obtained from the patient to publish this report in accordance with the journal's patient consent policy.

## Data Availability

The data that support the findings of this study are available from the corresponding author upon reasonable request.
